# Signatures of the Adler–Bell–Jackiw chiral anomaly in a Weyl fermion semimetal

**DOI:** 10.1038/ncomms10735

**Published:** 2016-02-25

**Authors:** Cheng-Long Zhang, Su-Yang Xu, Ilya Belopolski, Zhujun Yuan, Ziquan Lin, Bingbing Tong, Guang Bian, Nasser Alidoust, Chi-Cheng Lee, Shin-Ming Huang, Tay-Rong Chang, Guoqing Chang, Chuang-Han Hsu, Horng-Tay Jeng, Madhab Neupane, Daniel S. Sanchez, Hao Zheng, Junfeng Wang, Hsin Lin, Chi Zhang, Hai-Zhou Lu, Shun-Qing Shen, Titus Neupert, M. Zahid Hasan, Shuang Jia

**Affiliations:** 1 International Center for Quantum Materials, School of Physics, Peking University, Beijing, China; 2 Laboratory for Topological Quantum Matter and Spectroscopy (B7), Department of Physics, Princeton University, Princeton, New Jersey 08544, USA; 3 Wuhan National High Magnetic Field Center, Huazhong University of Science and Technology, Wuhan 430074, China; 4 Centre for Advanced 2D Materials and Graphene Research Centre, National University of Singapore, Singapore 117546, Singapore; 5 Department of Physics, National University of Singapore, Singapore 117542, Singapore; 6 Department of Physics, National Tsing Hua University, Hsinchu 30013, Taiwan; 7 Institute of Physics, Academia Sinica, Taipei 11529, Taiwan; 8 Condensed Matter and Magnet Science Group, Los Alamos National Laboratory, Los Alamos, New Mexico 87545, USA; 9 Department of Physics, University of Central Florida, Orlando, Florida 32816, USA; 10 Collaborative Innovation Center of Quantum Matter, Beijing 100871, China; 11 Department of Physics, South University of Science and Technology of China, Shenzhen, China; 12 Department of Physics, The University of Hong Kong, Pokfulam Road, Hong Kong, China; 13 Princeton Center for Theoretical Science, Princeton University, Princeton, New Jersey 08544, USA

## Abstract

Weyl semimetals provide the realization of Weyl fermions in solid-state physics. Among all the physical phenomena that are enabled by Weyl semimetals, the chiral anomaly is the most unusual one. Here, we report signatures of the chiral anomaly in the magneto-transport measurements on the first Weyl semimetal TaAs. We show negative magnetoresistance under parallel electric and magnetic fields, that is, unlike most metals whose resistivity increases under an external magnetic field, we observe that our high mobility TaAs samples become more conductive as a magnetic field is applied along the direction of the current for certain ranges of the field strength. We present systematically detailed data and careful analyses, which allow us to exclude other possible origins of the observed negative magnetoresistance. Our transport data, corroborated by photoemission measurements, first-principles calculations and theoretical analyses, collectively demonstrate signatures of the Weyl fermion chiral anomaly in the magneto-transport of TaAs.

## Abstract (web summary)

Anomalous conducting behavior of solids may reflect the presence of novel quantum states. Here, Zhang *et al*. report an increased conductivity in TaAs with a magnetic field applied along the direction of the current, which reveals an inherent property of the Weyl Fermion.

## Graphical abstract

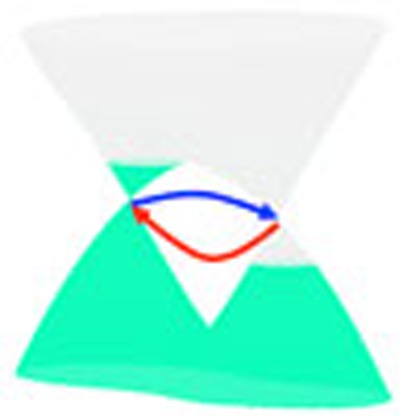

The principles of physics rest crucially on symmetries and their associated conservation laws. Over the past century, physicists have repeatedly observed the violations of apparent conservation laws in particle physics, each time leading to new insights and a refinement of our understanding of nature. One of the most interesting phenomena of this type is the breaking of a conservation law of classical physics by quantum-mechanical effects, a so-called anomaly in quantum field theory^1^. Perhaps the most primitive example is the so-called chiral anomaly associated with Weyl fermions^2^,^3^,^4^,^5^,^6^. A Weyl fermion is a massless fermion that carries a definite chirality. Due to the chiral anomaly, the chiral charge of Weyl fermions is not conserved by the full quantum-mechanical theory. Historically, the chiral anomaly was crucial in understanding a number of important aspects of the standard model of particle physics. The most well-known case is the triangle anomaly associated with the decay of the neutral pion *π*^0^ (refs 3, 4). Despite having been discovered more than 40 years ago, it remained solely in the realm of high-energy physics.

Recently, there has been considerable progress in understanding the correspondence between high-energy and condensed matter physics, which has led to deeper knowledge of important topics in physics such as spontaneous symmetry breaking, phase transitions and renormalization. Such knowledge has, in turn, greatly helped physicists and materials scientists to better understand magnets, superconductors and other novel materials, leading to important practical device applications. Here, we present the signatures of the chiral anomaly in a low-energy condensed matter Weyl system. In order to measure the chiral anomaly in a solid-state system, one needs to find a perturbation that couples differently to the two Weyl fermions of opposite chiralities. This is most naturally realized in a Weyl semimetal, in which the two Weyl cones are separated in momentum space. Recent theoretical and experimental advances have shown that Weyl fermions can arise in the bulk of certain novel semimetals with nontrivial topology^7^,^8^,^9^,^10^,^11^,^12^,^13^,^14^,^15^,^16^. A Weyl semimetal is a bulk crystal whose low-energy excitations satisfy the Weyl equation. Therefore, the conduction and valence bands touch at discrete points, the Weyl nodes, with a linear dispersion relation in all three momentum space directions moving away from the Weyl node. The nontrivial topological nature of a Weyl semimetal guarantees that Weyl fermions with opposite chiralities are separated in momentum space (Fig. 1a), and host a monopole and an antimonopole of Berry flux in momentum space, respectively (Fig. 1b). In this situation, parallel magnetic and electric fields can pump electrons between Weyl cones of opposite chirality that are separated in momentum space (Fig. 1a). This process violates the conservation of the the chiral charge, meaning that the number of particles of left and right chirality are not separately conserved^5^,^17^,^18^,^19^,^20^,^21^,^22^,^23^,^24^,^25^,^26^, giving rise to an analogue of the chiral anomaly in a condensed matter system. Apart from this elegant analogy and correspondence between condensed matter and high-energy physics, the chiral anomaly also serves as a crucial transport signature for Weyl fermions in a Weyl semimetal phase. Furthermore, theoretical studies have recently suggested that it has potential applications^27^.

In this paper, we perform magneto-transport experiments on the Weyl semimetal TaAs^12^,^13^,^14^,^16^. We observe a negative longitudinal magnetoresistance (LMR) in the presence of parallel magnetic and electric fields, which is indicative of the chiral anomaly due to Weyl fermions. On the other hand, due to the complicated nature of the magnetoresistence^28^,^29^,^30^,^31^,^32^,^33^,^34^,^35^,^36^,^37^,^38^, an unambiguous demonstration of the chiral anomaly remains lacking despite the volume of works reporting negative LMR^39^,^40^,^41^,^42^,^43^,^44^. Our data and careful analyses, which go beyond a simple observation of a negative LMR, allow us to systematically exclude other possible origins for the observed negative LMR. These data strongly support the chiral anomaly due to Weyl fermions in TaAs. Our studies demonstrate a low-energy platform where the fundamental physics of Weyl fermions and quantum anomalies can be studied in a piece of solid metal^17^,^18^,^19^,^20^,^21^,^22^,^23^,^24^,^25^,^26^,^27^.

## Results

### ARPES band structure

**Figure 1:**
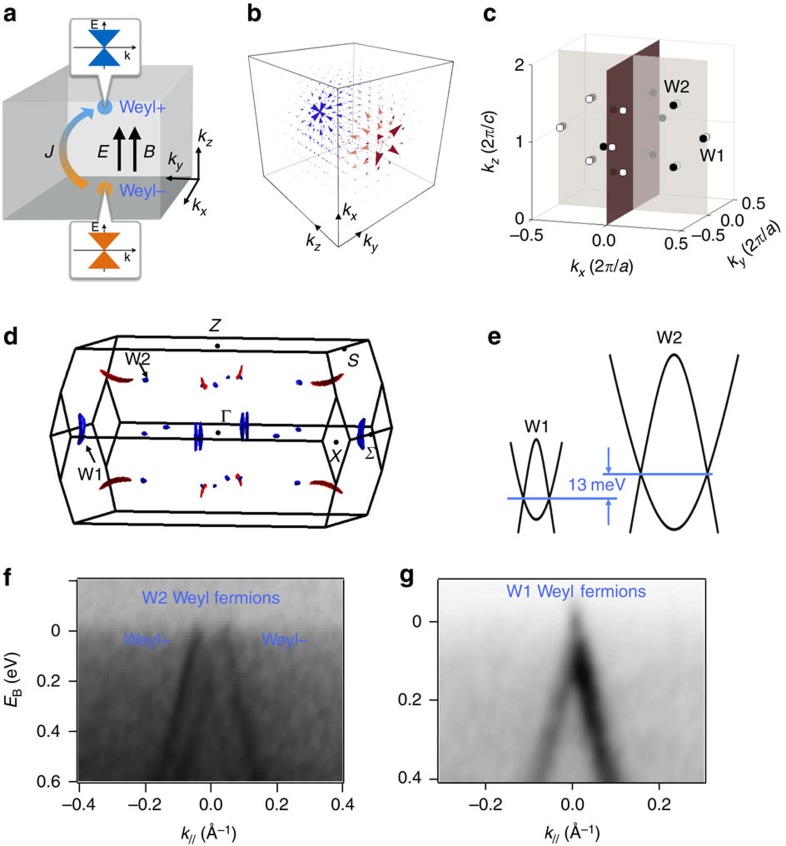
Electronic band structure of the Weyl semimetal TaAs. (**a**) Schematics of the separation of the pairs of Weyl fermions in a Weyl semimetal with opposite chiralities in momentum space, which is a direct consequence of its nontrivial topological nature. (**b**) Distribution of the Berry curvature near two Weyl nodes in momentum space with the opposite chiralities. (**c**) The location of the Weyl nodes in the first Brillouin zone. (**d**) First-principles calculated constant energy contour of TaAs. The energy is set at about 5 meV above the energy of the W2 Weyl nodes. (**e**) Schematic energy dispersions of the W1 and the W2 Weyl cones. (**f**) ARPES measured energy dispersions of the W2 Weyl cones. (**g**) ARPES measured energy dispersions of the W1 Weyl cones.

We start by presenting the key aspects of the bulk band structure of TaAs both in theory and in experiment. According to our first-principles calculation^13^,^14^, in total there are 24 bulk Weyl cones. We denote the 8 Weyl nodes that are located on the 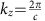 as W1 and the other 16 nodes that are away from this plane as W2 (Fig. 1c). There is a 13 meV offset between the energies of the W1 and W2 Weyl nodes (Fig. 1e). The pockets that arise from the Weyl fermions are shown in blue in Fig. 1d. Apart from the Weyl cones, there are additional (non-Weyl) hole-like bands crossing the Fermi level shown by the red ring-shaped contours in Fig. 1d.

We independently study the bulk electronic structure via angle-resolved photoemission spectroscopy (ARPES). This is important because relying entirely on numerical band structure calculations is not conclusive. Particularly, numerical band calculations have little power in predicting the position of the chemical potential of real samples, which is crucial for transport experiments. Figure 1f shows an *E*−*k*_||_ dispersion map that cuts across the two nearby W2 Weyl cones. The dispersion map reveals two linearly dispersive bands. The *k*-space distance between the two crossing points is about 0.08 Å^−1^, consistent with the calculated results. More importantly, our ARPES measurement shows that the native chemical potential of the samples is very close to the energy of the W2 Weyl nodes. Our data also reveals the W1 Weyl cones. As shown in Fig. 1g, the energy of the W1 Weyl node is below that of the W2 Weyl node (Fig. 1f), which is consistent with band calculation results. Systematic ARPES data can be found in the Supplementary Fig. 1 and Supplementary Note 1. We also observe the trivial hole bands in ARPES. The essential observations are listed as follows. There are three types of bands at the Fermi level, the W1 and W2 Weyl nodes and a trivial hole-like band. The native chemical potential is close to the energy of the W2 Weyl nodes, which is 13 meV higher than that of the W1 Weyl nodes. Therefore, the W1 Weyl cones form electron-like pockets, the trivial hole-like bands form hole-like pockets, and the W2 Weyl cones have low-carrier concentration, which can be electron- or hole-like depending on the specific position of the native chemical potential with respect to the W2 Weyl node in each sample batch.

### Quantum oscillation data

**Figure 2:**
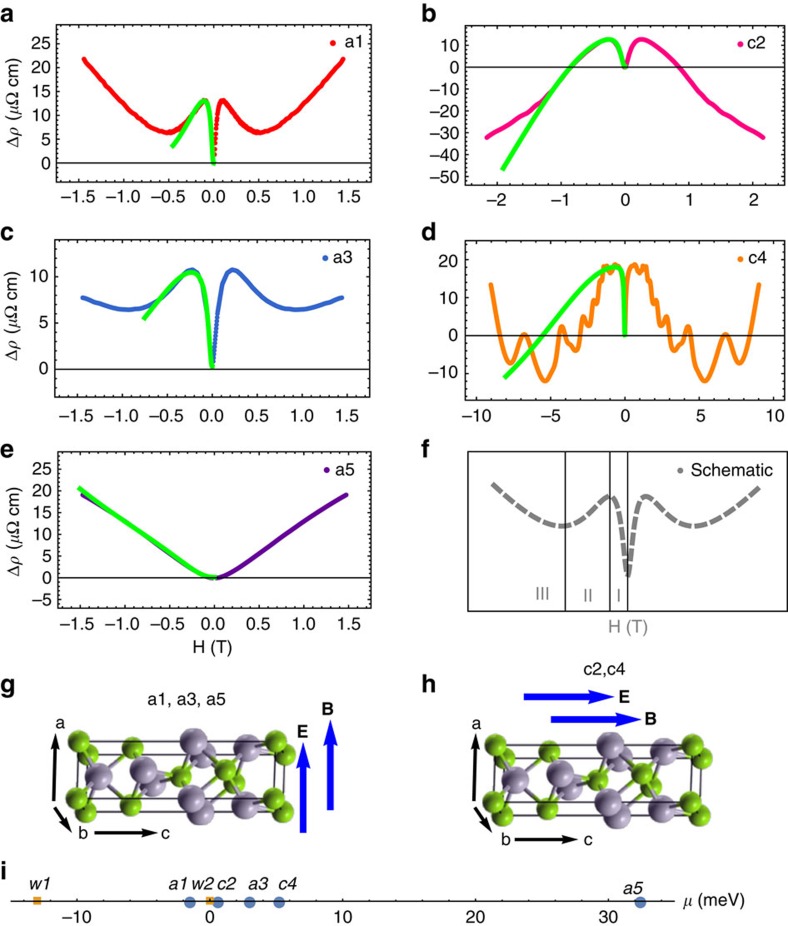
Observation of negative longitudinal magneto-resistances. (**a**–**e**) LMR data at *T*=2 K for samples a1, c2, a3, c4 and a5, respectively. The green curves are the fits to the LMR data in the semiclassical regime. The *y* axes of **a**–**e** are the change of the resistivity with respect to the zero-field resistivity, Δ*ρ*=*ρ*(*B*)−*ρ*(*B*=0). (**h**) A schematic drawing of the LMR data to show the three important features (I–III) observed in our data as a function of the magnetic field. (**g**) Measurement geometry for samples a1, a3 and a5. (**h**) Measurement geometry for samples c2 and c4. (**f**) A schematic illustration of the LMR data. The data consists of three sections as a function of the magnetic field, which are labled as I–III. (**i**) Position of the samples' chemical potential with respect to the energy of the Weyl nodes obtained from SdH oscillation measurements.

We have performed magneto-transport measurements on our TaAs samples, in order to probe the band structure at the Fermi level (Supplementary Fig. 2 and Supplementary Note 2). Our Hall data indeed reveal a coexistence of electron and hole carriers. We obtained critical band parameters, such as Fermi wavevector, Fermi velocity, chemical potential, carrier mobilities and so on, from the Shubnikov de-Haas (SdH) oscillation data. All band parameters obtained from the SdH oscillations are consistent with first-principles calculation and ARPES results. Most importantly, this enables us to determine the position of the chemical potential with respect to the Weyl nodes, as shown in Fig. 2i. We name the samples by a letter (a or c) followed by a number (1–5). The letter ‘a' or ‘c' means that the electrical current is along the crystallographic *a*- or *c*-axes. The number ‘1–5' refers to a sequence of the samples' chemical potential with respect to the energy of the W2 Weyl node from below the node to above the node (Fig. 2i). We also provide the Fermi energy of the TaAs samples determined by different approaches in Supplementary Note 3 and Supplementary Table 1.

### Longitudinal magnetoresistance

We now present our LMR data, without a pre-biased assumption of their origin. Figure 2a–e show the LMR data of five different batches of samples. The LMR data show three main features as a function of the magnetic field, as schematically drawn in Fig. 2f. At very small fields close to *B*=0, we observe a sharp increase of the LMR. Following the sharp increase, the LMR is found to decrease in an intermediate *B* field range. This is the negative LMR. While further increasing the *B* field, the LMR starts to increase again. We note that because features I and II likely have independent origins, the LMR is not necessarily absolutely negative (we do, however, observe absolute negative LMR in samples c2 and c4). Hence, more precisely, we speak of negative LMR in this paper, if the resistivity decreases with an increasing *B* field. In addition to these general features, we observe other more sample-dependent features: for sample c4, our data show clear quantum oscillations at a quite wide *B* field range of 0.5 T≤*B*≤8 T. For other samples, the quantum oscillations are much weaker but they are still visible. For sample a5, the LMR increases monotonically as a function of the *B* field. No negative LMR is observed.

**Figure 3:**
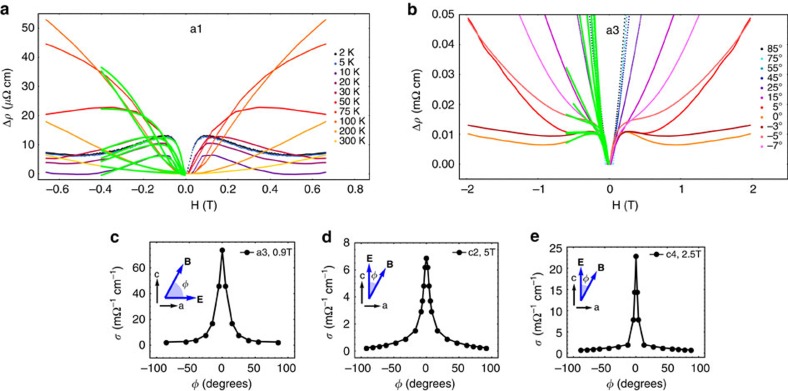
Systematic dependence of the negative longitudinal magneto-resistances. (**a**) Temperature-dependent LMR data for sample a1. (**b**) Magnetoresistance data as a function of the angle between the 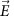 and 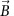 fields. The green curves of **a**,**b** are the fits to the LMR data in the semiclassical regime. The *y* axes are the change of the resistivity with respect to the zero-field resistivity, Δ*ρ*=*ρ*(*B*)−*ρ*(*B*=0). (**c**–**e**) The magnetoresistance as a function of the angle for samples c2, a3 and c4 at a fixed field.

We study the systematic dependence of the LMR on different parameters, including temperature, the angle between the 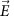 and 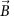 fields, and the direction of the current with respect to the crystallographic axis. The temperature-dependent data are shown in Fig. 3a for sample a1. Most notably, the negative LMR (feature II) shows a strong temperature dependence. At higher temperatures, for example, *T*≥50 K, the negative LMR vanishes. The dependence on the angle between the 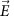 and 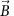 fields are shown in Fig. 3b–e. Our data show that the negative MR exhibits a very strong angular dependence. It becomes quickly suppressed as one varies the direction of the magnetic 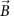 field away from that of the electric 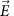 field. The dependence on the direction of the current with respect to the crystallographic direction is presented in Fig. 2. The measurements were performed with current along the crystallographic *a* axis for samples a1, a3 and a5, and with current along the *c* axis for samples c2 and c4. In both cases, the negative LMR is observed except for sample a5, whose chemical potential is far away from the energy of the Weyl nodes (Fig. 2i).

### Origins of the negative LMR

**Figure 4:**
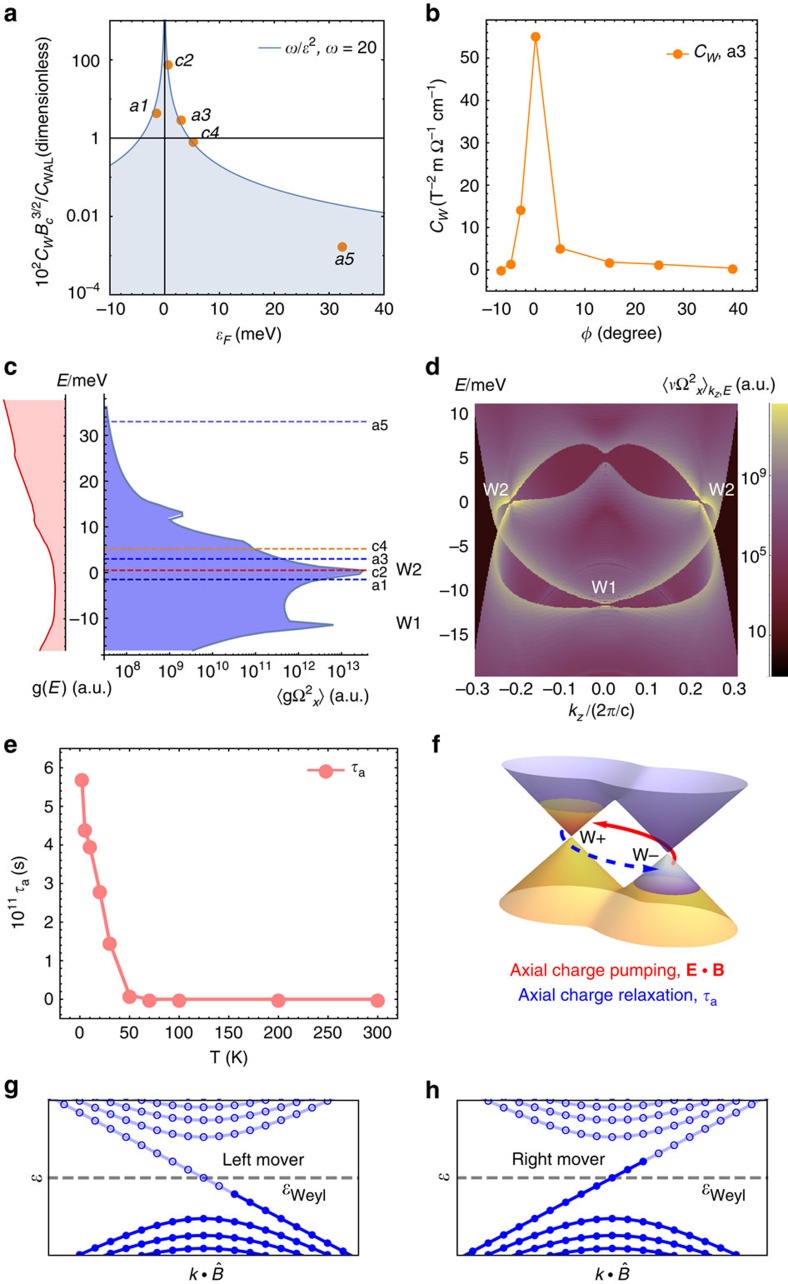
Signatures of the chiral anomaly due to Berry curvature of the Weyl fermions. (**a**) Chemical potential *E*_F_ dependence of the chiral coefficient *C*_W_. We expect the chiral coefficient *C*_W_ to decay as a function of 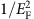. (**b**) Angle (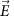 versus 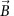) dependence of the chiral coefficient *C*_W_. (**c**) Density of states (*g*(*E*)) of the bulk electronic structure of TaAs shows a slow variation as a function of energy. The Berry curvature 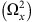 increases markedly at the energy close to the Weyl nodes. (**d**) Distribution of the square of the Berry curvature as a function of *k*_*z*_ and energy *E*, evidencing that the Weyl points are the dominant source of Berry curvature. The plot is integrated with respect to *kx* and *ky* over the whole Brillouin zone. (**e**)Temperature dependence of the axial charge relaxation time 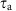 for sample a1. (**f**) A cartoon illustrating the chiral anomaly based on our LMR data. The chiral anomaly leads to the axial charge pumping, 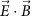. This causes a population imbalance difference between the Weyl cones with the opposite chiralities. The charge-pumping effect is balanced by the axial charge relaxation, characterized by the time scale 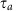 (refs 24, 26, 36). Note that the axial charge relaxation time 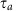 can be directly obtained from the observed negative LMR data through the chiral coefficient 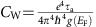. We also note that this is a cartoon that assumes the Fermi level at zero *B* field is exactly at the Fermi level. (**g**,**h**) Landau energy spectra of the left- and right-handed Weyl fermions in the presence of parallel electric and magnetic fields.

We now use these observations to understand the origin of the negative LMR. First, it is well-known that a negative LMR can arise in magnetic materials^28^. This obviously does not apply to our non-magnetic TaAs samples. The second possible origin is more classical due to geometry or size effects of the samples, such as the current-jetting effect^29^,^30^. These geometrical MR effects are also not consistent with our data, because they do not vanish quickly as one raises temperature^30^, and furthermore we have carefully shaped our samples to exclude the geometrical effects (Supplementary Fig. 3 and Supplementary Note 4). Third, we observe the negative LMR with current flowing both along the crystallographic *a*- and *c*-axes. We note that TaAs has a tetragonal lattice. Hence the *a-* and *c-*axes represent the largest anisotropy that the system could offer. The fact that the negative LMR is observed along both *a-* and *c*-axes proves that anisotropies in the system cannot explain our data^32^. Fourth, in the quantum limit, negative LMR can arise from the chiral, quasi one-dimensional character of the Landau levels that are formed by the band structure under magnetic fields. Essentially, it was predicted^31^,^33^ that a negative LMR can arise in any 3D metal irrespective of its band structure if the sample is in the ultra-quantum limit, which means that one has 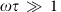 (*ω* is the cyclotron frequency and 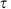 is the transport life time) and that the chemical potential only crosses the lowest Landau level (the Landau level index *N*=0, see Supplementary Fig. 4). This has been observed in doped semiconductor samples^35^. We have carefully checked whether our negative LMR is due to this mechanism. Particularly, one needs to be careful about the trivial hole-like bands in TaAs, because if they were in the ultra-quantum limit then it would have been entirely possible that the observed negative LMR were due to these trivial bands, rather than due to Weyl fermions in our samples. We note that the negative LMR are observed at small magnetic fields (for example, 0.1 T≤*B*≤0.5 T for sample a1). We have checked the 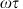 and the Landau level index *N* of our samples quantitatively (Supplementary Table 2), and our results show that all samples are always in the semiclassical limit at the small magnetic fields where the negative LMR are observed. Therefore, our data are inconsistent with this origin^31^,^33^. Fifth, a recent theoretical work has predicted a linear B-dependent magneto-conductivity in small fields^45^. However, this is also inconsistent with our data because predicted linear B-dependent magneto-conductivity requires the system to lie in the ultra-quantum limit. That is, only the lowest Landau band crosses the Fermi level, which is clearly not the case for our systems under study. Finally, in the semiclassical limit, nonzero LMR can arise from finite Berry curvature, as follows from the semiclassical equations of motion. Having excluded all other possibilities, we are led to conclude that our observed negative LMR must have this origin. However, as suggested in ref. 36 in addition to nonzero Berry curvature, an approximately conserved chiral charge density with long relaxation time—as found in Dirac and Weyl semimetals—is required to yield a negative LMR. The observed negative LMR is most likely to be attributed to Weyl nodes, in accordance with the theoretical analysis of (refs 24, 26 and 36). To confirm this picture independent of the assumption of effective low-energy Weyl Hamiltonians, we have studied the contribution of Berry curvature from each band carefully in a first-principles-derived model for TaAs (see Fig. 4c,d and Supplementary Fig. 5 and Supplementary Note 5). Our results show that in our TaAs system the Berry curvature almost entirely arises from the Weyl cones.

### The chiral anomaly

With such a conclusion, we are entitled to fit our LMR data with a semiclassical magnetoconductance formula that includes the contribution from Weyl nodes due to their Berry curvature. Specifically, we use the following equation.


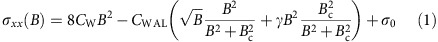


All coefficients are positive. The first term *σ*^chiral^=*C*_W_*B*^2^ is due to the Weyl fermions and will lead to a *B*^2^-dependent negative LMR. This term was systematically studied by transport theories in refs 24, 26. The chiral coefficient is 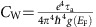 (refs 24, 26), where *g*(*E*_F_) is the density of states at the Fermi level, 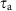 is the axial charge relaxation time and the additional factor of 8 is because we have 8 pairs of W2 Weyl nodes. All remaining terms contribute to positive LMR in the semiclassical regime. The *C*_WAL_ term arises from the 3D weak anti-localization (WAL) effect of the Weyl cones, which accounts for the initial steep uprise of the LMR at small magnetic fields. The 3D WAL is known to have a−*B*^2^ dependence near zero field and 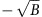 dependence at higher fields^46^. So we include a critical field *B*_c_ that characterizes a crossover. For the four samples a1, c2, a3, c4, the increase of the LMR at small magnetic fields are 230, 5, 156 and 47% compared to the zero-field resistance. Particularly, the increase for samples a1 and a3 is larger than 100%, which is usually not expected from the WAL scenario. On the other hand, we do notice that the increase is quite sample dependent, and that a similarly large increase (∼100%) of the magnetoresistance has also been reported in a concurrent transport work on TaAs^40^. In this work, we fit this initial uprise of the LMR by the WAL effect, but the anomalously large increase in samples a1, a3 and also in ref. 40 remains an theoretically open question that needs further investigation, which does not affect our main conclusion, that is, signatures of the chiral anomaly. Finally, the *σ*_0_ term is the positive LMR that arises from the Drude conductivity of conventional charge carriers present in TaAs. In parallel fields, the Lorentz force is zero so the Drude conductivity is a constant. More systematic details regarding the fitting are presented in Supplementary Fig. 6 and Supplementary Note 6.

The fitting results are shown by the green curves in Figs 2a–e and 3a,b. It can be seen that the fitting works well for the small *B* field region which includes the negative LMR. This is reasonable because the fitting formula is derived in the semiclassical limit. The angle dependence of the chiral coefficient *C*_W_ is shown in Fig. 4b for sample a3, which demonstrates that *C*_W_ is only significant in the presence of parallel electric and magnetic fields. The sharp angular dependence is an open theoretical problem. More importantly, we study the chemical-potential dependence of the LMR data. Our fitting captures quantitatively the relative size of the low-field positive LMR and the higher-field negative LMR as a function of chemical potential. We plot this ratio as a dimensionless quantity in Fig. 4a. We find that despite the simple form of the fitting formula, the different measurement geometries for the different samples, the presence of large quantum oscillations in sample c4 and large differences in the absolute resistivities of different samples, the chiral anomaly ratio scales as 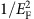. It is remarkable that this fitting result matches the simplest theoretical model for a Weyl point, where the Berry curvature 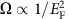. We emphasize that this provides powerful evidence that the negative LMR is due to the Weyl fermions. Note that the specific expression of the chiral coefficient, 
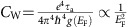
, is a result of the linear dispersion and the specific Berry curvature distribution of the Weyl cones (see Fig. 1b). Especially, at the energy of the W2 Weyl nodes, the trivial hole bands do not have any singularity. Thus if the negative LMR arose from the hole bands, then the chiral coefficient *C*_W_ would not have increased markedly as the chemical potential approaches the energy of the W2 Weyl nodes (Fig. 4a). Therefore, the obtained 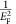 dependence of the chiral coefficient (Fig. 4a) provides a unique demonstration that our negative LMR is due to the Weyl fermions, because the 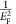 dependence reveals the details of the band dispersion and Berry curvature distribution of the Weyl cones, not just the fact that the bands have some nonzero Berry curvature. We use the above data and analyses to further exclude the possibility of negative LMR due to weak localization arising from the intervalley scattering. Our data is not due to the weak localization for the following reasons: First, weak localization does not have a strong *E*_F_ dependence, let alone the marked 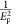 dependence observed in our data. Second, it has been theoretically shown that the magneto-conductivity without the chiral anomaly is always monotonic, even though the intervalley scattering can induce a negative LMR arising from the weak localization^46^. This is not consistent with our data, which means that, without the chiral anomaly, only weak localization/anti-localization cannot explain our data.

Up to here, we have demonstrated that the negative LMR arises from the nonzero Berry curvature of the Weyl fermions in our TaAs samples. We now establish the connection between our data and the chiral anomaly, the non-conservation of the electron quasi-particle number of the Weyl cones with a given chirality. In a real Weyl semimetal sample, this can be understood by two crucial components, the axial charge-pumping effect and the axial charge relaxation, as schematically shown in Fig. 4f. The charge-pumping effect means that a nonzero **E**·**B** can pump charges from one Weyl cone to the other, leading to an imbalance of the quasi-particle number of the Weyl cones with the opposite chiralities. This effect is well-established to occur between Weyl nodes of different chiral charge^24^,^26^,^36^, which are monopoles of Berry field strength in momentum space. We have directly shown the nontrivial Berry curvature monopoles associated with the Weyl fermions via our LMR transport data. The axial charge pumping creates an out-of-equilibrium quasi-particle distribution between the Weyl cones with opposite chiralities. To form a steady state, it is counteracted by the relaxation of the axial charge disproportionation through scattering between the Weyl nodes. The relaxation is characterized by a time scale, the axial charge relaxation time 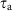. From our negative LMR data, we directly obtain the axial charge relaxation time 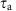 (Fig. 4e). The nonzero axial charge relaxation time 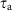 not only directly demonstrates the axial charge relaxation, but also confirms the existence of the axial charge pumping because these two are directly coupled, which means that one cannot exist alone if the other is absent.

We can directly obtain the axial charge relaxation time 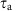, which serves as the critical physical quantity that characterizes the chiral anomaly, from the chiral coefficient *C*_W_ using the relationship 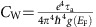 (refs 24, 26). In Fig. 3a, we present fitting results as a function of temperature for sample s1. We use the fitting coefficients *C*_W_ to obtain the axial charge relaxation time as a function of temperature, presented in Fig. 4e. We find that the 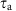 rapidly decays to zero with increasing temperature. This decay of 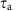 corresponds to the decay of the negative LMR with increasing temperature in the raw data and is expected because scattering typically increases with temperature. We obtain an axial charge relaxation time 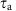
=5.96 × 10^−11^ s for sample a1 at *T*=2 K (Fig. 4e). Note that this 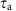 is associated with the W2 Weyl cones because the Fermi level is very close to the W2 nodes. On the other hand, it is difficult to obtain the transport life time of the W2 Weyl cones because the density of states at the Fermi level is dominated by contributions from the W1 Weyl cones and the trivial hole bands (Fig. 1d). Therefore, we estimate the quasi-particle life time associated with the W2 Weyl cones via 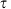
≃*ħ*/*E*_F_=7.04 × 10^−13^ s for sample a1. We see that the axial charge relaxation time 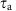 is much longer than the quasi-particle life time 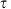. The imbalance of population due to the axial charge pumping can be also estimated by the uncertainty principle Δ*μ*=*ħ/*
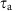
≃0.011 meV. At *E*_F_=−1.5 meV, the density of states per W2 Weyl cone is *g*(*E*_F_)=1.6 × 10^16^ states/(eV cm^−3^). Therefore, we estimate the chiral charge, the non-conservation of the quasi-particle number of the Weyl cone with a given chirality, to be Δ*μ* × *g*(*E*_F_)=1.6 × 10^14^. This directly characterizes the chiral anomaly in our Weyl semimetal TaAs sample.

## Discussion

We emphasize the critical logical sequences that are key to our demonstration. Unlike previous studies, we do not assume that the negative LMR arises from the chiral anomaly^40^,^41^,^42^,^43^,^44^. To demonstrate the chiral anomaly, it is critically important to consider all possible origins for a negative LMR and to discuss how one can distinguish each of the other origins from the chiral anomaly. We first excluded the geometry and spin(magnetic) effects. Then we show that our observed LMR is not in the quantum (large *B* field) limit, in which the Fermi energy crosses only the lowest Landau level. This is important because the LMR in the quantum (large *B* field) limit can be negative or positive depending on specific scenarios, such as the band dispersion and nature of the impurities^31^,^33^,^34^. In fact, it is even theoretically shown that the Weyl cones that respect time-reversal symmetry can contribute a positive (not a negative) LMR in the quantum limit if the field dependence of the scattering time and Fermi velocity of the Landau bands is fully respected^34^. Therefore, observing a negative LMR in the large-field quantum limit may not be a compelling signature of Weyl fermions. In the semiclassical (small *B* field) limit, after excluding the geometry and magnetic effects, one can avoid ambiguities in the physical interpretation since a negative LMR can only arise from a nonzero Berry curvature^24^,^26^,^36^,^37^. In fact, it has been shown that the LMR from a band with zero Berry curvature will always be positive^37^. However, we emphasize that, at a qualitative level, the negative LMR in the semiclassical limit is only a signature of the Berry curvature but it is not unique to Weyl fermions. In order to uniquely attribute the negative LMR to Weyl fermions, we discovered here that it is crucial to obtain comprehensive information about the band structure. Specifically, first we have shown that the Berry curvature in our TaAs is dominated by the Weyl cones. Second, the chiral coefficient has a 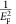 dependence. These two pieces of evidence, together with the full systematics of the data sets uniquely presented here, provides strong signatures of the chiral anomaly of Weyl fermions.

## Methods

### Sample growth and electrical transport

High-quality single crystals of TaAs were grown by the standard chemical vapour transport method as described in ref. 47. TaAs crystals were structurally characterized by powder X-ray diffraction to confirm bulk quality, and to determine (001) crystal face. A small portion of the obtained samples were ground into fine powders for X-ray diffraction measurements on Rigaku MiniFlex 600 with Cu *K*_*α*_ (40 kV, 15 mA; *λ*=0.15405, nm) at room temperature, and then refined by a Rietica Rietveld program. Magneto-transport measurements were performed using a Quantum Design Physical Property Measurement System. High-field electrical transport measurements were carried out using a pulsed magnet of 50 ms in Wuhan National High Magnetic Field Center. All the measurements were carried out from −9 to 9 T or −56 to 56 T.

### Angle-resolved photoemission spectroscopy

The soft X-ray ARPES (SX-ARPES) measurements were performed at the ADRESS Beamline at the Swiss Light Source in the Paul Scherrer Institut in Villigen, Switzerland using photon energies ranging from 300 to 1,000 eV (ref. 48). The sample was cooled down to 12 K to quench the electron–phonon interaction effects reducing the *k*-resolved spectral fraction. The energy and angle resolution was better than 80 meV and 0.07°, respectively. Vacuum ultraviolet ARPES measurements were performed at beamlines 4.0.3, 10.0.1 and 12.0.1 of the Advanced Light Source at the Lawrence Berkeley National Laboratory in Berkeley, California, USA, Beamline 5–4 of the Stanford Synchrotron Radiation Light source at the Stanford Linear Accelerator Center in Palo Alto, California, USA and Beamline I05 of the Diamond Light Source in Didcot, UK, with the photon energy ranging from 15 to 100 eV. The energy and momentum resolution was better than 30 meV and 1% of the surface Brillouin zone.

### Theoretical calculations

First-principles calculations were performed by the OPENMX code within the framework of the generalized gradient approximation of density-functional theory^49^. Experimental lattice parameters were used^47^, and the details for the computations can be found in our previous work in ref. 13. A real-space tight-binding Hamiltonian was obtained by constructing symmetry-respecting Wannier functions for the As *p* and Ta *d* orbitals without performing the procedure for maximizing localization, similar for calculations done for topological insulators^50^,^51^.

## Additional information

**How to cite this article:** Zhang, C.-L. *et al.* Signatures of the Adler–Bell–Jackiw chiral anomaly in a Weyl fermion semimetal. *Nat. Commun.* 7:10735 doi: 10.1038/ncomms10735 (2016).

## Supplementary Material

Supplementary Information Supplementary Figures 1-6, Supplementary Tables 1-2, Supplementary Notes 1-7 and Supplementary References
