# Supplementary material for: Signatures of the Adler–Bell–Jackiw chiral anomaly in a Weyl fermion semimetal
Source: Nat Commun. 2016 Feb 25;7:10735. doi: 10.1038/ncomms10735 (PMC4773426; doi:10.1038/ncomms10735)
Supplement: Supplementary Information — Supplementary Figures 1-6, Supplementary Tables 1-2, Supplementary Notes 1-7 and Supplementary References [file ncomms10735-s1.pdf]

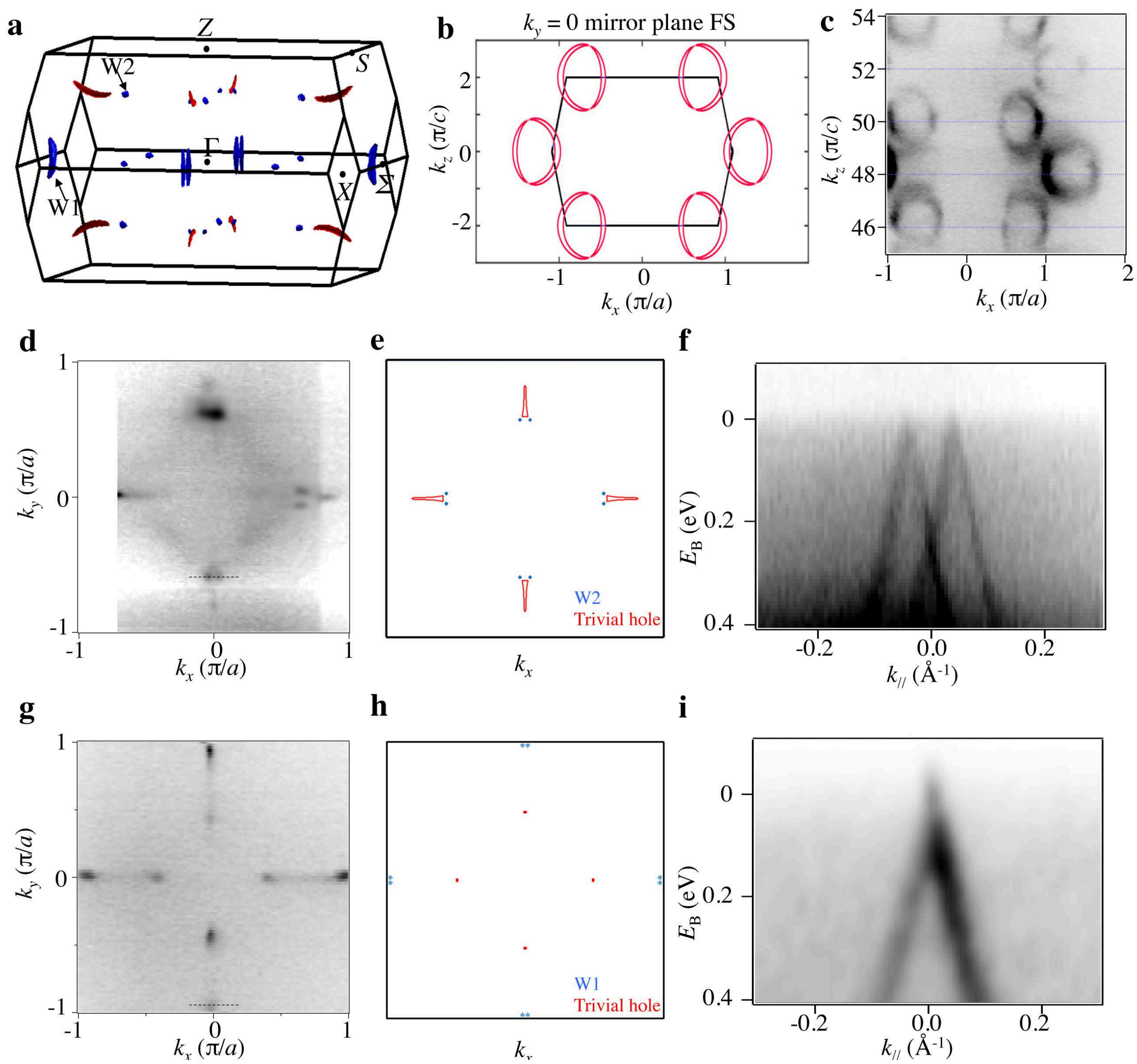

Supplementary Figure 1: **Systematic angle-resolved photoemission data. a,** First-principles calculated bulk Fermi surface in k_x, k_y, k_z space. **b,c,** Calculated and ARPES measured bulk Fermi surface in k_x, k_z space on the $k_y = 0$ plane, showing the constant energy contours that arise from the trivial hole-like bands. **d,e,** ARPES measured and calculated bulk Fermi surface in k_x, k_y space at the k_z value that corresponds to the W2 Weyl nodes. **f,** ARPES $E - k_{//}$ dispersion map revealing the two nearby W2 Weyl cones. The direction of the dispersion cut is shown by the dotted line in panel (e). **g-i,** Same as panels (**d-f**) but for the W1 Weyl cones.

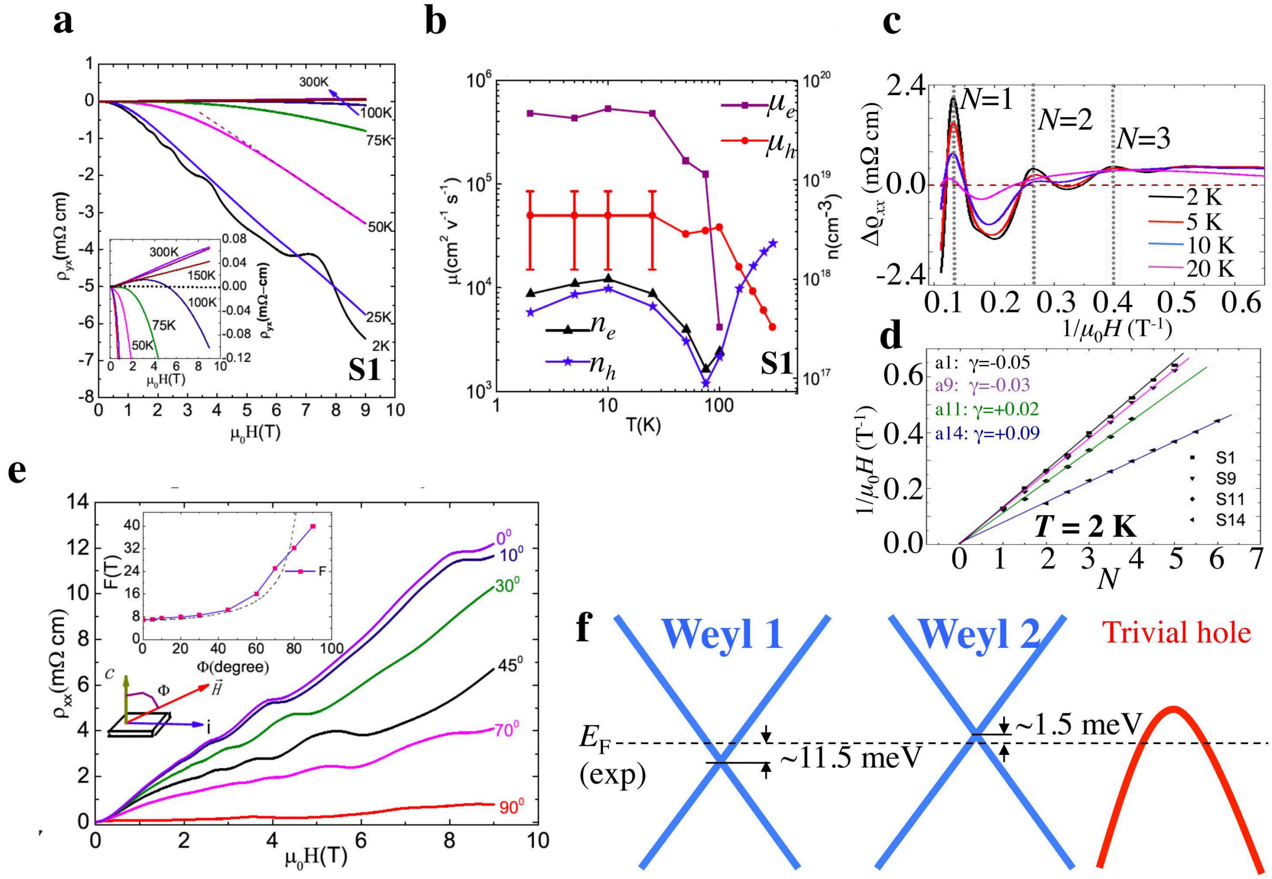

Supplementary Figure 2: **Magneto-Transport and quantum oscillation data. a,** Hall resistivity versus the magnetic field in the temperature range from 2 to 300 K. Strong SdH oscillations were observed at 2 K. Inset: the high temperature Hall resistivity. **b,** Mobilities and carrier concentrations of the electrons and holes, clearly showing the coexistence of Weyl electrons and trivial holes in our samples. **c,** The oscillatory parts of σ_{xx} at various temperatures, showing the π Berry's phase of the Weyl electron pocket. **d,** The SdH fan diagram for four different samples. All of the four intercepts are located around zero, suggesting the π Berry's phase of the Weyl electron pocket **e,** Magnetic field dependence of resistivity at representative Φ angles between 0° - 90° at 2K for sample a1, after heating the sample. The MR decreases rapidly when the magnetic field is tilted from c to the direction of the current i. Inset: the frequency F versus Φ. The dashed curve is $(1/\cos\Phi)\cdot F_0$. **f,** Schematics of the experimentally determined band diagram indicating the positions of the two different types of Weyl nodes, W1 and W2, as well as the trivial hole-like band, relative to the experimentally determined Fermi level of sample a1.

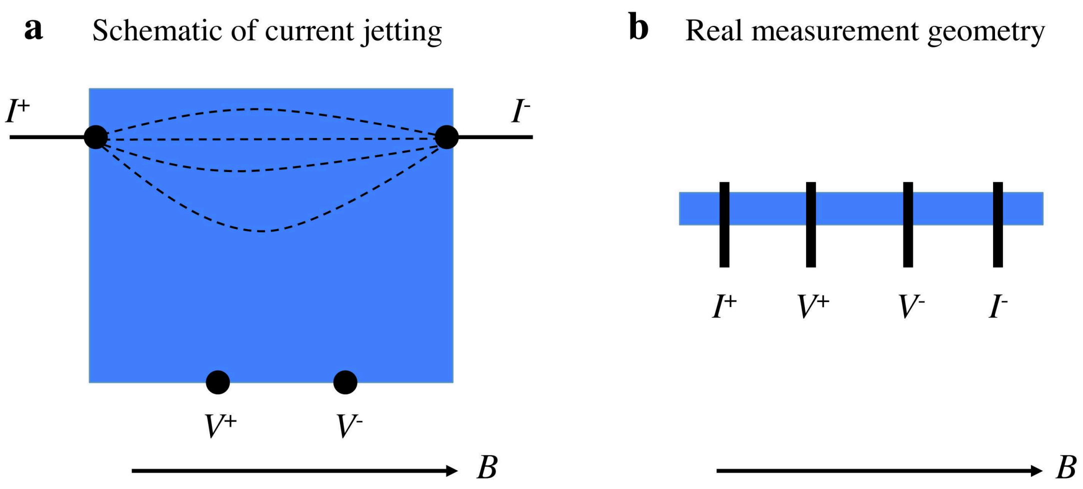

Supplementary Figure 3: **a,** A schematic illustration of the current jetting effect [12]. **b,** Our samples are carefully shaped to exclude the geometry/size effects.

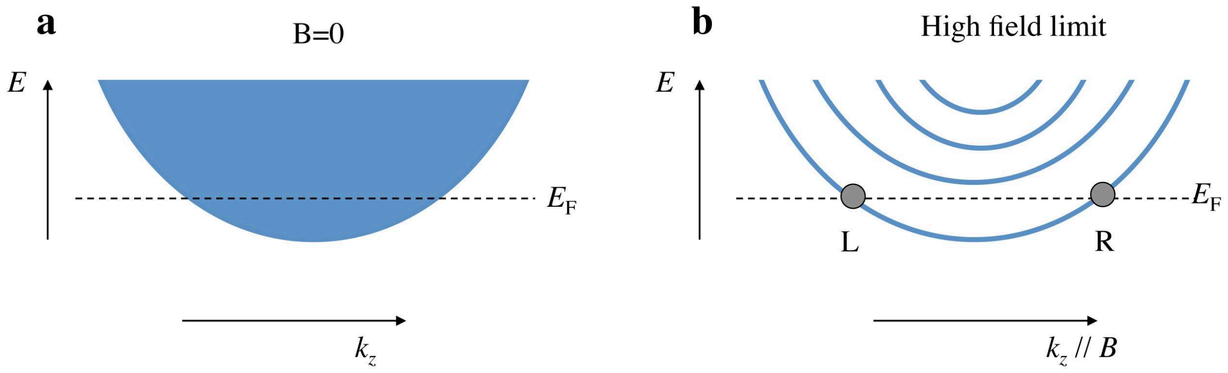

Supplementary Figure 4: **a**, Schematic band structure of a generic 3D metal without magnetic field. **b**, Schematic band diagram in the quantum limit regime.

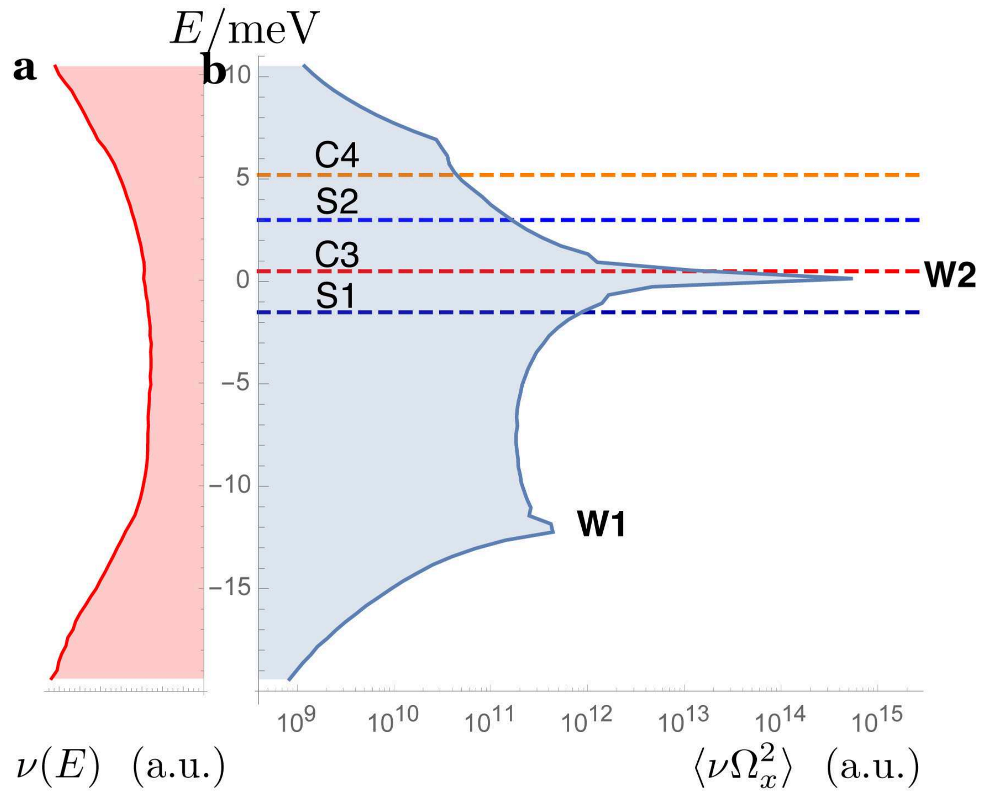

Supplementary Figure 5: **a,** Density of states of the bulk band structure of TaAs. **b,** Average of the square of the Berry curvature Ω_1 over surfaces of equal energy in the bulk band structure of TaAs. The results were obtained using an effective $k \cdot p$ Hamiltonian that was fitted to the DFT band structure. While the density of states is featureless, the averaged Berry curvature is strongly enhanced near the Weyl nodes W1 and W2. This proves that the Weyl cones really dominate the contribution to the Berry curvature.

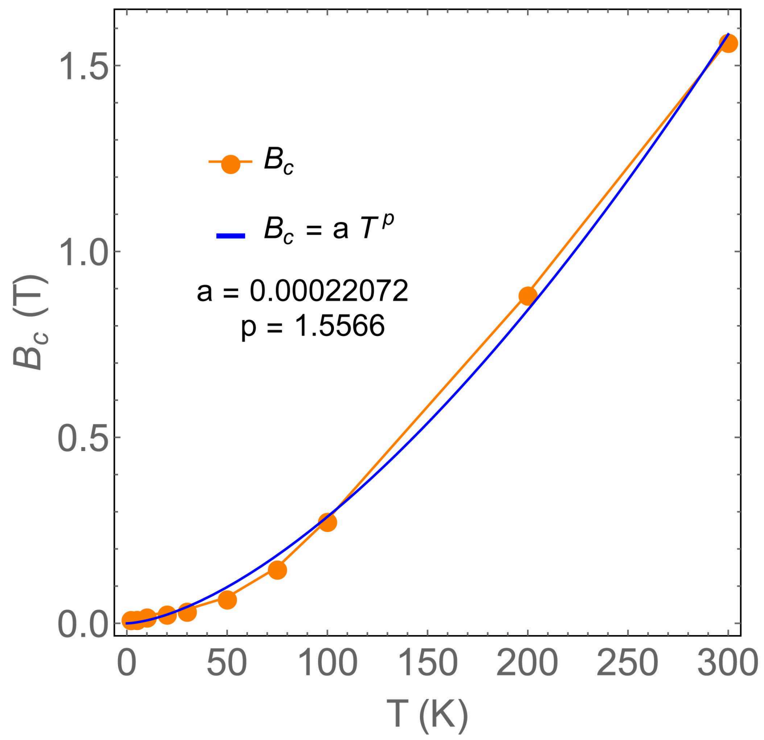

Supplementary Figure 6: B_c as a function of temperature obtained from fitting of the temperature dependent LMR data for sample a1 (Fig. 3a in the main text).

DFT calculation	ARPES	Transport
-8 meV	-5 ± 1.5 meV	Sample a1: -1.5 ± 0.86 meV
		Sample c2: 0.61 ± 0.18 meV
		Sample a3: 3 ± 1.23 meV
		Sample c4: 5.22 ± 0.58 meV
		Sample a5: 32.41 ± 0.83 meV

Supplementary Table 1: **Fermi energy ($E_\mathbf{F}$) with respect to the W2 Weyl node.**

	$\omega\tau$	LL index N
Sample a1	1.2	115
Sample c2	0.06	51
Sample a3	0.3	30
Sample c4	0.19	18

Supplementary Table 2: **Band parameters for the trivial hole pockets.** The $\omega\tau$ and Landau level index N parameters of the trivial hole bands obtained from our transport measurements. These parameters are determined at the magnetic field of 0.3 T, which is a representative field value where the negative LMR takes place.

Supplementary Note 1

In this section, we present systematic ARPES data to reveal the bulk electronic structure of TaAs. Our ARPES data show that there are three pockets that cross the Fermi level, namely, W1 Weyl cones, W2 Weyl cones and the trivial hole pockets. Supplementary Figure 1**c** shows the ARPES Fermi surface on the $k_y = 0$ mirror plane. We observe ring-shaped hole-like contours. These contours demonstrate the trivial hole pockets, consistent with the calculations (Supplementary Figure 1**c**). In Supplementary Figure 1**d**, we show ARPES measured k_x, k_y Fermi surface at the k_z value that corresponds to the W2 Weyl nodes. We observe that the Fermi surface consists of discrete points, which are the W2 Weyl nodes. The energy dispersion measurement in Supplementary Figure 1**f** indeed reveals two cones, which are the two nearby W2 Weyl cones. Similarly, Figs. 1**g-i** are data showing the W1 Weyl cones and Weyl nodes. We find that the energy of the W1 Weyl node is about 10-20 meV lower than that of the W2 Weyl node (Figs. 1**f,i**), in agreement with the calculation (13 meV). More precisely determining their energy difference is limited by the experimental energy resolution (> 40 meV). Because the k-space splitting of the W1 Weyl nodes is much smaller than that of the W2, resolving the two nearby W1 Weyl cones is beyond the experimental resolution. We emphasize that our ARPES data are important in the following aspects. First, our ARPES data experimentally prove the existence of Weyl fermions in our TaAs samples. Second, they experimentally reveal that there are three kinds of pockets crossing the Fermi level, W1 Weyl cones, W2 Weyl cones and the trivial hole bands. Third, they experimentally determine the Fermi energy, which is quite close to the W2 Weyl nodes. Thus W1 Weyl cones contribute electron pockets and the trivial bands contribute hole pockets.The W2 Weyl cones have small carrier density, and can be either electron-like or hole-like depending on the specific position of the Fermi energy with respect to the W2 nodes. These observations are crucial for our transport measurements.

Supplementary Note 2

We carried out magneto-transport and quantum oscillation measurements to determine the band parameters of the TaAs samples in our transport experiments. In Figure 2, we show the magneto-transport data on sample a1 as a representative example. Same measurements

were also performed on other samples presented in the main text in order to obtain their band parameters. Figure 2**a** shows the Hall resistance ρ_{yx} of sample a1 at different temperatures, clearly revealing a coexistence of the electron and hole carriers. This is consistent with the ARPES and first principles results presented above. We obtain the carrier density n and the mobility μ for the electron and hole carriers, shown in Supplementary Figure 2**b**.

We analyze the SdH quantum oscillation data at $T = 2$ K. We use the following expression to analyze the SdH oscillation data, ρ_{xx} at $T = 2$ K, for a 3D system [5].

$$\rho_{xx} = \rho_0[1 + A(B,T)\cos 2\pi(F/B + \gamma)] \qquad \text{(Supplementary Equation 1)}$$

$$A(B,T) \propto exp(-2\pi^2 k_B T_D/\hbar\omega_c)\frac{2\pi^2 k_B T/\hbar\omega_c}{sinh(2\pi^2 k_B T/\hbar\omega_c)}, \qquad \text{(Supplementary Equation 2)}$$

Here, ρ_0 is the non-oscillatory part of the resistivity, $A(B,T)$ is the amplitude of the SdH oscillations, B is the magnetic field, γ is the Onsager phase, T_D is the Dingle temperature, k_B is the Boltzmann's constant, ω_c is the cyclotron frequency, and $F = \frac{\hbar}{2\pi e}A_{\mathrm{F}}$ is the frequency of the oscillations. A_{F} is the extremal cross-sectional area of the Fermi surface (FS) associated with the Landau level (LL) index N, e is the electron charge, and $2\pi\hbar$ is the Planck's constant. For sample a1, we obtain a Fermi surface area of $A_{\mathrm{F}} = 7.07 \times 10^{-4}$ $\mathrm{\AA}^{-2}$ and a Fermi wave vector k_{F} is $\sqrt{A_{\mathrm{F}}/\pi} = 0.015$ $\mathrm{\AA}^{-1}$. We note that since the magnetic field is parallel to the c crystallographic axis, the obtained Fermi surface area corresponds to the 2D cross-section of the 3D Fermi pocket that is perpendicular to the k_z direction.

The Landau level index N is plotted as a function of the inverse of the magnetic field strength $(1/\mu_0 H)$ in Supplementary Figure 2**d**, from which one can see that, for all four samples, the linear interpolation of the curve intersects with the x near zero ($\gamma = 0$). This suggests that the electron carriers arise from a linearly dispersive band with a non-trivial Berry's phase [5], consistent with the ARPES and first principles results that the electron carriers mainly arise from the W1 Weyl cones.

In order to obtain the Fermi velocity, the energy position of the chemical potential, and other important band parameters, we apply Supplementary Equation 2. We obtained a cyclotron mass m_{cyc} of $0.15m_{\mathrm{e}}$. We obtain the Fermi wave vector k_{F} is $\sqrt{A_{\mathrm{F}}/\pi} = 0.015$ $\mathrm{\AA}^{-1}$, and the Fermi velocity v_F is $\hbar k_{\mathrm{F}}/m_{\mathrm{cyc}} = 1.16 \times 10^5$ m/s. Using a linear dispersion of this electron pocket, we obtain the chemical potential (relative to the energy of the W1

Weyl node) to be $E_{\mathrm{F}} = m_{\mathrm{cyc}} v_{\mathrm{F}}^2 = 11.48$ meV for sample a1. Note that different samples have slightly different values for the chemical potential and other band parameters. This variation helps us to study the systematic dependence of the negative LMR, which is crucial for understanding its origin.

To further confirm that the observed electron carriers indeed arise from the W1 Weyl cones, we study the anisotropy of the electron-like pocket by tilting the magnetic field away from the c direction in sample a1. Our data (Supplementary Figure 2**e**) shows that the Fermi surface area along the a axis is about 5 times larger that of along the c axis. Therefore, the transport data show that the electron-like Fermi pocket is an ellipsoid that is elongated along the c axis. We systematically check if the obtained band parameters from transport are consistent with ARPES and calculation. We place the chemical potential at 11.48 meV above the Weyl nodes W1 in our first-principles calculations and try to compare the calculated band parameters to those of obtained from transport. We have found an excellent agreement between calculation and transport: (1) The calculated carrier density of the electron pockets is 5.07×10^{17} cm^{-3}, which agrees with our experimentally measured value in Supplementary Figure 2**d**. (2) The anisotropy of the Fermi surface area is found to be 4.9, which is also in line with the experimentally determined value of 5.

Based on our systematic measurements, we obtain a band diagram presented in Supplementary Figure 2**f**. The chemical potential lies ~ 11.5 meV above W1. Therefore it is very close to W2, consistent with the ARPES results shown above.

Supplementary Table 2 shows the $\omega\tau$ (ω is the cyclotron frequency and τ is the transport lifetime) and the Landau level index N parameters of the trivial hole bands determined from our transport measurements. These parameters define the (semiclassical or quantum) regime. Both $\omega\tau$ and ν are a function of the external magnetic field. The values in Supplementary Table 2 is obtained at the magnetic field of 0.3 T, which is a representative field value where the negative LMR takes place. Specifically, $\omega\tau$ is obtained by the following relationship, $\omega\tau = \mu H$, where μ is the carrier mobility and H is the magnetic field. The Landau level index N is obtained from the Onsager relation (Supplementary Equation 3).

$$S_F(B) = \frac{2\pi e B}{\hbar}(N + \gamma), \qquad \text{(Supplementary Equation 3)}$$

It can be seen that for the magnetic field of 0.3 T, none of the samples satisfy $\omega\tau >> 1$ and Landau level index $N = 0$ (E_{F} only crosses the lowest LL). Thus the hole bands are not in

the quantum limit. This is reasonable because the negative LMR is observed at quite low field (e.g. 0.3 T).

In order to extract the information of the carriers, the Hall conductivity tensor $\sigma_{\rm xy} = \rho_{\rm yx}/(\rho_{\rm xx}^2 + \rho_{\rm yx}^2)$ was fitted by adopting a two-band model derived from the two-band theory [2],

$$\sigma_{xy} = [n_h\mu_h^2\frac{1}{1+(\mu_h H)^2} - n_e\mu_e^2\frac{1}{1+(\mu_e H)^2}]eH, \qquad \text{(Supplementary Equation 4)}$$

Where n_e (n_h) and μ_e (μ_h) denote the carrier concentrations and mobilities for the electrons (holes), respectively. Since there are four free parameters in this formula, we applied two constraints in the fitting process [3]. The first constraint is the zero field resistivity.The second constraint we adopted is the Hall resistivity in the large B-field limit. In high field, the Hall resistivity reads $\rho_x y = 1/ec \times 1/(n_e - n_h) \times B$, so we can find the value of $n_e - n_h$ by a linear fitting of the high field data. These two constraints are standard constraints in transport works, as reviewed in Ref. [3]. Therefore, the total number of free parameters in fits was be reduced to two.

Supplementary Note 3

In the Supplementary Table 1, we provide the Fermi energy of the TaAs samples determined by different approaches.

The "+" and "−" sign in the table means the $E_{\rm F}$ being above or below the energy of the W2 Weyl node, respectively.The DFT calculated Fermi level is the norminal Fermi level directed obtained from the DFT calculation. The ARPES Fermi level was obtained by a linear fitting of the low energy Weyl fermion dispersion data (e.g. Fig. 1a). The transport Fermi level data was obtained from the SdH oscillation data. We note that the Fermi energy of the sample a5 is quite different from the others because sample a5 was grown by a different method where a different agent was used in the chemical vapor transport growth process. The error bars in this table are the errors in the fitting process.

Supplementary Note 4

We provide a diagrammatical highlight of the logical sequence for the experiments.

Step-0 Basic spirit: Magnetoresistance is a quite complicated phenomenon [6–19]. A negative LMR by itself, although quite rare, is not a unique signature of the Weyl fermions [6–19].

In all previous studies [20–25] reporting the chiral anomaly in Weyl and Dirac semimetals, the authors started by assuming that the negative LMR arises from the chiral anomaly. However, the key aspect that was missed is that it is entirely possible that these systematic dependence can be also consistent with other origins of the negative LMR. Specifically, none of the works have listed all possible origins for a negative LMR, and none of the works have discussed how one can distinguish each of the other origins from the chiral anomaly.

This is what has been achieved in this paper. We have considered comprehensively the possible origins for a negative LMR, and we have presented systematic data and analyses that lead us to the unique conclusion of the chiral anomaly due to Weyl fermions. In the process of doing that, we found that not only systematic LMR data (temperature, angular and other dependences) are necessary, but also having comprehensive information about the band structure at the Fermi level is crucial. Below we elaborate on the logical sequence of our work.

Step-1 Negative LMR in magnetic element based materials: The origin that is the easiest to exclude is the negative MR in magnetic materials, such as the giant magnetoresistance and the colossal magnetoresistance [6–11]. These effects do not strongly depend on the angle between the electrical $\vec{E}$ and the magnetic $\vec{B}$ fields and occur in both the transverse MR and longitudinal MR. This is not consistent with our observations because our TaAs sample is nonmagnetic and because the MR in our experiment is only negative in the presence of parallel electrical $\vec{E}$ and magnetic $\vec{B}$ fields.

Step-2 Classical (extrinsic) geometry or size effects: A negative LMR can arise from a number of classical (extrinsic) geometry or size effects. They have been explained in detail in Ref. [12]. Essentially, these effects are caused by the inhomogeneous spatial distribution of the current in the sample. For example, Supplementary Figure 3**a** shows a schematic of a scenario where the current jetting effect can take place. Because the current and voltage contacts are misaligned in a four-probe setting, the current is largely distorted to be localized in the upper part of the sample when the current i is parallel to the magnetic field H (as indicated by the dotted lines). This causes a decrease of the measured voltage,

leading to a negative LMR [12]. Other geometry or size effects are quite similar. For example, in polycrystalline samples, the inhomogeneity of the sample can also distort the current leading to a negative LMR. Similarly, anisotropy of the sample can cause similar effects.

In order to exclude the geometry and size effects, the samples were shaped into long, thin bars (thickness < 100 μm) with four silver paste contacts fully crossing their width (Supplementary Figure 3**b**). This precaution can effectively prevent the current from distorting. Moreover, we measured many samples with different sizes (thickness). All samples with different thickness exhibited comparable negative LMR. This is inconsistent with the size or geometry effect, which is expected to depend strongly on the size and the shape of the samples. Furthermore, we have shown that the observed negative LMR is completely suppressed at temperatures above 50 K. By contrast, the negative LMR from geometry or size effects were found to survive even at room temperatures [13]. We also note that we observe the negative LMR with current flowing both along the crystallographic a and c axes. We note that TaAs is a tetragonal lattice. Hence the a and c axes represent the largest anisotropy that the system can offer. The fact that the negative LMR is observed along both a and c axes proves that it is irrelevant to the anisotropy of the system.

Step-3 Chiral Landau levels in the quantum limit: It has been theoretically pointed out [14, 16] that a negative LMR can occur in a general 3D metal if the system is in the quantum limit regime, meaning that (1) one has $\omega\tau >> 1$ (ω is the cyclotron frequency and τ is the transport lifetime) and that (2) the chemical potential only crosses the lowest Landau level. This is quite intuitive because in the ultra quantum limit, the system essentially has a pair of 1D chiral fermions with the opposite chiralities that arise from the lowest Landau level (Supplementary Figure 4**b**). Apparently, this construction [14, 16] does not depend on the band structure details and can be realized in a generic 3D metal in the ultra quantum limit.

In order to check whether our negative LMR is due to this mechanism, we have carefully studied in which regime (semiclassical or quantum) our samples are located at the magnetic fields corresponding to the negative LMR. We note that the negative LMR are observed in low fields (e.g. $0.1\ \mathrm{T} \leq B \leq 0.5\ \mathrm{T}$ for sample a1). We have checked it quantitatively (see Table 2). For all samples studied, the system is always in the semiclassical limit.

We further emphasize the tricky nature of the LMR in the quantum (large B field) limit. First, as mentioned above, Ref. [16] showed that it is possible for a negative LMR to occur in a general 3D metal in the quantum limit. Second, Refs. [16, 17] showed that the sign of the LMR in fact depends on the nature of the impurities in the quantum limit [16, 17]. In fact, it is even theoretically shown that the Weyl cones that respect time-reversal symmetry can contribute a positive (not a negative) LMR in the quantum limit if the field dependence of the scattering time and Fermi velocity of the Landau bands is fully respected [17]. Therefore, the sign of the LMR in the quantum regime seems to depend on the details of the impurities and scattering mechanisms. It cannot provide strong evidence for the Weyl fermions or the chiral anomaly.

Step-4 Berry curvature in the semiclassical limit: In the semiclassical limit, a negative LMR can occur if the band structure has a nonzero Berry curvature at the Fermi level [19]. The negative LMR due to Weyl fermions belongs to this case because Weyl nodes serve as sources or drains of Berry curvature. However, it is also important to note that theoretically any band with a nonzero Berry curvature will contribute a term in the equation of motion that leads to a negative LMR [19]. This means that, even up to here, after we have excluded many origins above, we can show that the observed LMR is due to the nonzero Berry curvature of the band structure in the semiclassical limit. We cannot yet show that it is uniquely due to the Weyl fermions, let alone the chiral anomaly.

Thus we further show two pieces of evidence to establish the connection between the negative LMR and the Weyl fermions in TaAs. (1) We have studied the contribution of Berry curvature from each bands carefully (see Supplementary Figure 5). We show that in our TaAs system the Berry curvature almost entirely arises from the Weyl cones. The contribution from the trivial hole like bands is negligible. (2) We have fitted the negative LMR and found that the chiral coefficient C_{W} has a $\frac{1}{E_{\mathrm{F}}^2}$ dependence. Note that the $\frac{1}{E_{\mathrm{F}}^2}$ dependence of the chiral coefficient C_{W} is a result of the linear dispersion and the specific Berry curvature distribution of the Weyl cones. In order words, if one started with a different band (not Weyl) that has a different dispersion and a different distribution of the Berry curvature, then the expression of the chiral coefficient would have been different and the $C_{\mathrm{W}} \propto \frac{1}{E_{\mathrm{F}}^2}$ dependence would have been invalid. These data and analyses show that the observed negative LMR is not due to the trivial hole bands. Instead, it arises from the Weyl

fermions in TaAs.

We emphasize that this last step is crucial. One of the reasons that this can be achieved in our study is that we have systematically mapped out the band structure using three independent ways (first-principles, ARPES and quantum oscillations). All the other systematic dependences, including the ($\vec{E}$ vs $\vec{B}$) angle, temperature, and the current direction with respect to the crystallographic axis, which are presented here and also in Ref. [20–25], can ***not*** distinguish the negative LMR due to Weyl fermions from the negative LMR due to other band structures with nonzero Berry curvature. In other words, if a negative LMR were induced by a (non-Weyl) band structure due to its nonzero Berry curvature, then it will also show quantitatively the same angular, temperature, and current direction dependences. Therefore, we emphasize that it is crucial to have full information of the band structure of the system studied in transport. Also, the $\frac{1}{E_{\mathrm{F}}^2}$ dependence of the chiral coefficient C_{W}, which is uniquely presented here not in other studies, is crucial because it really depends on the details of the band dispersion and Berry curvature distribution of the Weyl cones, not just the fact that the bands have some nonzero Berry curvature.

Supplementary Note 6

In order to calculate the Berry curvature of the band structure, we built a $k \cdot p$ model whose general structure was introduced in Ref. [1]. In the absence of spin-orbit coupling, the Hamiltonian H_0 has a nodal ring in the ($k_2 = 0$) plane that is protected by mirror symmetry. When spin-orbit coupling is included, the degeneracy of the nodal ring is lifted by the mass terms $m_i,\ i = 1, \cdots, 6$ that contribute to H_{mass} below. The combinations of these mass terms can give rise to pairs of Weyl nodes off the ($k_2 = 0$) plane.

A pair of Weyl nodes in the ($k_3 = 0$)-plane are generated by the combination of the masses m_4 and m_6, while the Weyl nodes away from the ($k_3 = 0$)-plane stem from the interplay of m_4 and m_5.

The total Hamiltonian is given by

$$H = \quad H_0 + H_{\text{mass}}, \qquad \text{(Supplementary Equation 5)}$$

$$H_0 = \quad \epsilon(\mathbf{k})\sigma_0 + d_1(\mathbf{k})\sigma_1 + d_2(\mathbf{k})\sigma_2 + d_3(\mathbf{k})\sigma_3, \qquad \text{(Supplementary Equation 6)}$$

$$H_{\text{mass}} = \quad m_1(\mathbf{k})\sigma_0 s_2 + m_2(\mathbf{k})\sigma_3 s_2 + m_3(\mathbf{k})\sigma_1 s_1 + m_4(\mathbf{k})\sigma_1 s_3 + m_5(\mathbf{k})\sigma_2 s_3 + m_6(\mathbf{k})\sigma_2 s_1, \qquad \text{(Supplementary Equation 7)}$$

where $s_\mu,\ \mu = 0, \cdots, 3$, are the 2×2 identity matrix and the three Pauli matrices acting on the spin degree of freedom of the electrons and $\sigma_\mu,\ \mu = 0, \cdots, 3$, are the 2×2 identity matrix and the three Pauli matrices acting on an effective orbital degree of freedom.

The $k_2 \to -k_2$ mirror symmetry, as well as the combination of time-reversal and C_2 rotation symmetry, impose whether each term is even or odd under $k_2 \to -k_2$ and under $k_3 \to -k_3$. We expand each term up to a given order in $\mathbf{k}$, including only symmetry-allowed contributions:

$$\varepsilon(\mathbf{k}) = \mu + wk_1 + \mathcal{O}(\mathbf{k}^2), \qquad \text{(Supplementary Equation 8)}$$

$$d_1(\mathbf{k}) = uk_2k_3 + \mathcal{O}(\mathbf{k}^3), \qquad \text{(Supplementary Equation 9)}$$

$$d_2(\mathbf{k}) = vk_2 + \mathcal{O}(\mathbf{k}^2), \qquad \text{(Supplementary Equation 10)}$$

$$d_3(\mathbf{k}) = M - ak_1^2 - bk_3^2 + ck_1 + dk_1^3 + \mathcal{O}(\mathbf{k}^3), \qquad \text{(Supplementary Equation 11)}$$

$$m_1(\mathbf{k}) = m_1 + \mathcal{O}(\mathbf{k}), \qquad \text{(Supplementary Equation 12)}$$

$$m_2(\mathbf{k}) = m_2 + \mathcal{O}(\mathbf{k}), \qquad \text{(Supplementary Equation 13)}$$

$$m_3(\mathbf{k}) = m_3k_3 + \mathcal{O}(\mathbf{k}^2), \qquad \text{(Supplementary Equation 14)}$$

$$m_4(\mathbf{k}) = m_4 + m_4'k_1 + \mathcal{O}(\mathbf{k}^2), \qquad \text{(Supplementary Equation 15)}$$

$$m_5(\mathbf{k}) = m_5k_3 + \mathcal{O}(\mathbf{k}^2), \qquad \text{(Supplementary Equation 16)}$$

$$m_6(\mathbf{k}) = m_6 + \mathcal{O}(\mathbf{k}). \qquad \text{(Supplementary Equation 17)}$$

We then fitted this $k \cdot p$ Hamiltonian to the first-principle band structure with the goal to reproduce the ring-shaped trivial Fermi surface and the correct location and number of Weyl nodes. This is achieved with the parameters $M = 12.23$, $\mu = -3.504$, $u = -763.1$, $v = -685.1$, $w = 34.11$, $a = 682.8$, $b = 583.0$, $c = 264.2$, $d = -147.5$, $m_1 = 7.019$,

$m_2 = 1.031$, $m_3 = 0.9078$, $m_4 = 0.0$, $m_4' = -11.07$, $m_5 = -56.50$, $m_6 = -4.097$, all in units of meV and the appropriate power of Å.

This effective Hamiltonian was used to compute the Berry curvature $\Omega_i = \mathrm{i}\epsilon_{ijl}\langle\partial_{k_j}u(\mathbf{k})|\partial_{k_l}u(\mathbf{k})\rangle$, where $|u(\mathbf{k})\rangle$ are nondegenerate Bloch states. (For the isolated degeneracy points in the BZ we have to consider the non-Abelian form of the Berry curvature.)

Figure 5 shows the average of Ω_1^2 over a contour of equal energy E weighted with the density of states $\nu(E)$ at this energy. While the density of states is featureless, the averaged Berry curvature is strongly enhanced near the Weyl nodes W1 and W2. We clearly observe two peaks in $\langle\nu\Omega_1^2\rangle$ which stem from the dominant Berry curvature near the Weyl nodes W1 and W2 (notice the log scale). Figure 5 also shows that no other anomalous sources of diverging Berry curvature exist in the band structure, besides the Weyl nodes. (The actual divergence at the Weyl points is cut off due to the numerical accuracy.) This proves that the Weyl cones really dominate the contribution to the Berry curvature, and that the contribution from the trivial hole like bands is negligible.

Supplementary Note 6

As described in the main text, we use the following equation to fit the LMR data.

$$\sigma_{xx}(B) = 8C_{\mathrm{W}}B^2 - C_{\mathrm{WAL}}\left(\sqrt{B}\frac{B^2}{B^2+B_{\mathrm{c}}^2} + \gamma B^2\frac{B_{\mathrm{c}}^2}{B^2+B_{\mathrm{c}}^2}\right) + \sigma_0$$

(Supplementary Equation 18)

Here we discuss additional details regarding the fitting:

Firstly, we discuss the 3D weak anti-localization (WAL) term. The 3D WAL effect, the C_{WAL} term in the fitting formula, accounts for the initial steep uprise of the LMR at small magnetic fields. In the fitting formula, we have included a critical field B_{c} that characterizes the crossover from a $-B^2$ dependence near zero field to $-\sqrt{B}$ dependence at higher fields. Here we describe the underlying physical reason for this crossover carefully. B_c is related to the phase coherence length ℓ_ϕ. At low temperatures and no intervalley scattering, $\ell_\phi \to \infty$, theory predicts a $-\sqrt{B}$ dependence of the WAL [17]. This means that one has $B_c \simeq 0$. In the other limit, meaning high temperatures or in the presence of strong inter-valley scattering, $\ell_\phi \to 0$, and a $-B^2$ dependence is theoretically expected [17]. This means that B_c is large.

Roughly,

$$B_c \sim \frac{\hbar}{e\ell_\phi^2}. \qquad \text{(Supplementary Equation 19)}$$

Empirically, the temperature dependence of the phase coherence length can be written as $\ell_\phi \sim T^{-p/2}$, then $B_c \sim (\hbar/e)T^p$, where p is positive and determined by decoherence mechanisms such as electron-electron interaction ($p = 3/2$) or electron-phonon interaction ($p = 3$). Also, We expect that the intervalley scattering may correct ℓ_ϕ from these simple power law.

In Fig. 3a of the main text, we have presented the temperature dependent LMR data for sample a1 and their fits. Here in Supplementary Figure 6 we show the corresponding B_c as a function of temperature. It can be seen that at low temperatures the B_c is almost zero, which indicates that the WAL follows well the $-\sqrt{B}$ dependence. At higher temperature, B_c increases monotonically. We fit the B_c as a function of T, from which we extract that $p \approx 1.5$, indicating that the electron-electron interaction is the dominant decoherence mechanism.

Secondly, regarding the σ_0 term in the fitting formula (Supplementary Equation 18). We note that it contributes the positive LMR that arises from the Drude conductivity of conventional charge carriers present in TaAs. In parallel fields, the Lorentz force is zero so the Drude conductivity is a constant, under the assumption that the corresponding Fermi surface is isotropic. In the case of TaAs, the Fermi surface that gives rise to the positive LMR is the trivial hole pocket, which is quite anisotropic. Thus a weak magnetic field dependence can also be possible. However, we note that even if there is a weak magnetic field dependence for the Drude conductivity in parallel fields, it has to be positive because the only source of negative LMR in the semiclassical limit is the Berry curvature. And as we have shown clearly in the main text, in TaAs, the Berry curvature comes from the Weyl cones, not the trivial hole pockets. In our case, we find that using a constant for the Drude conductivity already gives satisfactory results for the fitting of the LMR data, which is sufficient for our purpose. This also reduces free parameters in the fitting, making the fitting more robust.

Finally, we note that except the chiral C_{W} term, all other terms in Supplementary Equation 18 give rise to positive LMR. They are used to simulate the other effects (Drude conductivity or WAL) that coexist with the chiral charge pumping. Therefore, they are not the focus of our paper. The focus is the chiral C_{W} term, which characterizes the chiral anomaly.

Supplementary Note 7

The y axes of Figs. 2**a-e** and Figs. 3**a-b** are the change of the resistivity with respect to the zero-field resistivity, $\Delta\rho = \rho(B) - \rho(B = 0)$. The zero-field resistivities are 5.65, 221.28, 7.02, 40.12, 12.59 (μOhm cm) for Figs. 2**a-e**, respectively; 5.65, 6.10, 12.59, 10.32, 12.44, 19.91, 31.90, 46.54, 87.07, 122.53 (μOhm cm) for temperature $T = 2$, 5, 10, 20, 30, 50, 75, 100, 200, 300 K in Fig. 3**a**, and 5.43, 12.04, 7.56, 6.13, 7.91, 9.02, 6.82, 7.02, 8.02, 5.91, 8.27 (μOhm cm) for angles 85, 75, 55, 40, 25, 15, 5, 0, -3, -5, -7 (degree) in Fig. 3**b**.

Correspondence and requests for materials should be addressed to M.Z.H. (Email: mzhasan@princeton.edu) and S.J. (gwljiashuang@pku.edu.cn).

[1] Weng, H., Fang, C., *et al.* Weyl semimetal phase in non-centrosymmetric transition metal monophosphides. *Phys. Rev. X* **5**, 011029 (2015).

[2] Colin, M. H. *The hall effect in metals and alloys.* Cambridge University Press (1972).

[3] Ando, Y. Topological Insulator Materials. *Journal of the Physical Society of Japan* **82**, 25 (2013).

[4] Liang, T. *et al.* Ultrahigh mobility and giant magnetoresistance in the Dirac semimetal Cd_3As_2. *Nature Mater.* **14**, 280-284 (2015).

[5] Murakawa, H. *et al.* Detection of Berry's Phase in a Bulk Rashba Semiconductor. *Science* **342**, 1490-1493 (2013).

[6] Fert, A. Nobel Lecture: Origin, development, and future of spintronics. *Rev. Mod. Phys.* **80**, 1517-1530 (2008).

[7] Searle, C. & Wang, S. Studies of the ionic ferromagnet (LaPb)MnO3 III. Ferromagnetic resonance studies. *Canadian journal of physics* **47**, 2703-2708 (1969).

[8] Searle C. & Wang, S. Studies of the ionic ferromagnet (LaPb)MnO3. V. Electric transport and ferromagnetic properties. *Canadian journal of physics* **48**, 2023-2031 (1970).

[9] Jin, S. *et al.* Thousandfold Change in Resistivity in Magnetoresistive La-Ca-Mn-O Films. *Science* **264**, 413-415 (1994).

[10] Taylor, G. R., Isin, A. & Coleman, R. V. Resistivity of Iron as a Function of Temperature and Magnetization. *Phys. Rev* **165**, 621-631 (1968).

[11] Ritchie, L. *et al.* Magnetic, structural, and transport properties of the Heusler alloys Co2MnSi and NiMnSb. *Phys. Rev. B* **68**, 104430 (2003).

[12] Pippard, A. B. *Magnetoresistance in metals.* Cambridge University Press, 1989.

[13] Hu, J., Rosenbaum, T. F. & Betts, J. B. Current Jets, Disorder, and Linear Magnetoresistance in the Silver Chalcogenides. *Phys. Rev. Lett.* **95**, 186603 (2005).

[14] Argyres, P. N. & Adams, E. N. Longitudinal Magnetoresistance in the Quantum Limit. *Phys. Rev.* **104**, 900-908 (1956).

[15] Kikugawa *et al.* Realization of the axial anomaly in a quasi-two-dimensional metal. Preprint at http://arxiv.org/abs/1412.5168 (2014).

[16] Goswami, P. Pixley, J. H. & Das Sarma, S. Axial anomaly and longitudinal magnetoresistance of a generic three dimensional metal. *Phys. Rev. B* **92**, 075205 (2015).

[17] Lu, H. -Z., Zhang, S. -B. & Shen, S. -Q. High-field magnetoconductivity of topological semimetals with short-range potential. *Phys. Rev. B* **92**, 045203 (2015).

[18] Sugihara, K., Tokumoto, M., Yamanouchi, C. & Yoshihiro, K. Longitudinal Magnetoresistance of n-InSb in the Quantum Limit. *J. Phys. Soc. Jpn.* **41**, 109-115 (1976).

[19] Burkov, A. A. Negative longitudinal magnetoresistance in Dirac and Weyl metals. *Phys. Rev. B* **91**, 245157 (2015).

[20] Kim, H.-J. *et al.* Dirac versus Weyl Fermions in Topological Insulators: Adler-Bell-Jackiw Anomaly in Transport Phenomena. *Phys. Rev. Lett.* **111**, 246603 (2013).

[21] Huang, X. *et al.* Observation of the chiral anomaly induced negative magneto-resistance in 3D Weyl semi-metal TaAs. Preprint at http://arxiv.org/abs/1503.01304 (2015).

[22] Zhang, C. *et al.* Observation of the Adler-Bell-Jackiw chiral anomaly in a Weyl semimetal. Preprint at http://arxiv.org/abs/1503.02630 (2015).

[23] Xiong, J. *et al.* Sinature of the chiral anomaly in a Dirac semimetal: a current plume steered by a magnetic field. Preprint at http://arxiv.org/abs/1503.08179 (2015).

[24] Zhang, C. *et al.* Detection of chiral anomaly and valley transport in Dirac semimetals. Preprint at http://arxiv.org/abs/1504.07698 (2015).

[25] Shekhar, C. *et al.* Large and unsaturated negative magnetoresistance induced by the chiral anomaly in the Weyl semimetal TaP. Preprint at http://arxiv.org/abs/1506.06577 (2015).
